# Between Two Grammatical Gender Systems: Exploring the Impact of Grammatical Gender on Memory Recall in Ukrainian−Russian Simultaneous Bilinguals

**DOI:** 10.1111/cogs.70117

**Published:** 2025-10-02

**Authors:** Oleksandra Osypenko, Silke Brandt, Panos Athanasopoulos

**Affiliations:** ^1^ Department of Linguistics and English Language Lancaster University; ^2^ Centre for Languages and Literature Lund University; ^3^ Department of General Linguistics Stellenbosch University

**Keywords:** Linguistic relativity, Grammatical gender, Cognitive processing, Memory, Simultaneous bilingualism

## Abstract

This study examines the impact of grammatical gender on memory recall among simultaneous bilinguals with two three‐gendered languages (Ukrainian and Russian). Ukrainian−Russian bilinguals and English monolingual controls were tested on their ability to remember names assigned to objects with either matching or mismatching grammatical genders across their two languages. Results showed that bilinguals recalled names more accurately when the biological sex of the names was congruent with the grammatical gender of objects in both languages (e.g., recalling a male name assigned to a noun with masculine grammatical gender in both L1s, rather than a female name). English monolinguals, in contrast, showed no difference in recall. However, when grammatical gender mismatched across Ukrainian and Russian, the expected influence of the more proficient language on recall accuracy was not observed. These findings suggest that converging grammatical information from two L1s creates stronger memory associations, enhancing recall accuracy of simultaneous bilinguals. Conversely, mismatching grammatical genders appear to negate this effect. Taken together, these findings highlight the interconnected nature of bilingual conceptual representation.

## 1 Introduction

The principle of linguistic relativity posits that the languages we speak influence our thoughts in systematic ways (Casasanto, 2008; Lucy, 1997; Whorf, 1956). Various disciplines (i.e., linguistics, philosophy, and psychology, as well as interdisciplinary research) have put this hypothesis at the forefront of their investigations. Research in linguistic relativity has explored multiple areas, including grammatical number and object perception, spatial‐temporal orientation, time, and grammatical gender. The current study focuses on grammatical gender in the Ukrainian and Russian languages and its effects on cognitive processes, specifically how grammatical gender influences memory recall. Grammatical gender has received extensive attention, with some studies affirming its effects (e.g., Boutonnet, Athanasopoulos, & Thierry, 2012; Sato, Casaponsa, & Athanasopoulos, 2020; Sato & Athanasopoulos, 2018) and others showing evidence against its effect on cognitive processes or proposing alternative explanations (e.g., Bassetti, 2007; Pavlidou & Alvanoudi, 2013; Sera et al., 2002).

In the present study, we focus on addressing two key gaps that we identified in linguistic relativity research on grammatical gender. First, we aim to draw attention to the inclusion of the relatively underrepresented group of simultaneous bilinguals. The need to consider multilingualism was arguably put forward in some of Whorf's arguments (see Pavlenko, 2016). However, when it comes to focusing on bilingual individuals, researchers tend to focus on sequential bilinguals (e.g., Boutonnet et al., 2012; Sato et al., 2020; Phillips & Boroditsky, 2003). Meanwhile, simultaneous bilinguals, who acquire both languages (L1 and a second L1, henceforth 2L1) from birth, are scarcely represented in the research (Bassetti, 2007; Osypenko, Brandt, & Athanasopoulos, 2025). Therefore, little is known about whether the cognition of adult simultaneous bilinguals with two distinct grammatical genders embedded in their L1 and 2L1 is affected by language to the same degree as sequential bilinguals. Second, early research on linguistic relativity (Sera et al., 2002; Vigliocco, Vinson, Paganelli, & Dworzynski, 2005) argued that language effects are present in speakers of two‐gendered languages rather than three‐gendered languages, as there is a stronger association with natural gender in the former group. Finally, when looking into the domain of grammatical gender, a large number of studies typically target categorization mechanisms with a prominent grammatical gender present, for example, the voice‐assignment task, where participants are asked to assign either a male or female voice to gendered objects (Kurinski, Jambor, & Sera, 2016; Sera et al., 2002). However, we intend to investigate the effects using a less gender‐salient paradigm that involves recall memory (Boroditsky & Schmidt, 2000).

Our study aims to address these issues by looking at two three‐gendered languages (Ukrainian and Russian) coexisting in the mind of simultaneous bilinguals. It will allow us to tackle another uncovered issue in linguistic relativity research: How do two grammatical systems that have been acquired since early childhood interact with each other? More importantly, are the effects of language on cognition enhanced when grammatical gender in L1 matches grammatical gender in 2L1? Alternatively, are the language effects negated or reduced when incorporating stimuli where grammatical gender in L1 mismatches with 2L1?

### 1.1 Effects of grammatical gender in linguistic relativity research in bilinguals

Grammatical gender is present in approximately 40% of the world's languages (Corbett, 2013), requiring speakers to mark gender through noun suffixes, as well as articles, adjectives, pronouns, and, in specific cases, within verb forms, in such languages as Ukrainian and Russian. This compels speakers of gendered languages to pay close attention to grammatical gender during language production and comprehension. This grammatical property has been extensively employed in linguistic relativity research for several reasons, including its cross‐linguistic variability and inherent arbitrariness (Everett, 2013; Boutonnet et al., 2012). For instance, the noun “sun” is grammatically feminine in German (“*die Sonne*”), masculine in Spanish (“*el sol*”), and neuter in both Ukrainian (“*сонце*”) and Russian (“*солнце*”), exemplifying the absence of any systematic relationship between grammatical gender and the semantic or biological attributes of the referent. Multiple studies with monolingual speakers provide evidence in favor of grammatical gender effects on cognitive processes (e.g., categorization) and conceptual representations of nouns (Haertlé, 2017; Maciuszek, Polak, & Świa̧tkowska, 2019; Vernich, 2017). A significant body of research has also investigated grammatical gender effects in bilingual speakers, specifically sequential bilinguals (Athanasopoulos & Boutonnet, 2016; Chen & Faitaki, 2024; Kurinski & Sera, 2011, among others). This allows for a deeper investigation of Whorfian effects, such as how two languages coexist in a bilingual mind, specifically whether bilinguals exhibit language effects comparable to monolingual speakers of their L1s or whether having two grammatical systems leads to differences in cognitive processes (Cook et al., 2006). Additionally, including bilinguals provides the opportunity to examine the stability of previously found gender effects. Specifically, testing bilinguals in their second language (L2) that does not have a specific grammatical/lexical property of their first language (L1), allows researchers to test whether the effects of L1's gender on bilinguals’ responses can remain despite the presence of an L2 (Kousta, Vinson, & Vigliocco, 2008; see Samuel et al., 2019 for a detailed review). Our study contributes to the growing body of research on grammatical gender effects in bilingual speakers by focusing on a relatively underexplored group within linguistic relativity studies—simultaneous bilinguals. These are individuals who acquire two languages from birth or very early in life and develop native‐like proficiency in both first languages (henceforth, both L1s). In our case, the participants are simultaneous bilinguals of Ukrainian and Russian, two languages with partially contrasting grammatical gender systems.

Both Ukrainian and Russian have a three‐gender grammatical system where all nouns are categorized as feminine, masculine, or neuter, which provides an interesting test‐case for this line of research. In these languages, animate nouns generally align with the biological sex of the referent, except for certain exceptions (e.g., “*мавпа*” (monkey) is feminine in Ukrainian regardless of gender, see Vakulenko, 2023). However, when the biological sex of the referent is unknown or irrelevant, speakers commonly use the default grammatical gender assigned to the noun ‐ for instance, “*слон* ” (elephant) is typically used with masculine gender in Ukrainian, while “*лисиця* ” (fox) takes feminine gender, regardless of the animal referent's actual sex (Vakulenko, 2023). Neuter forms also occur, most notably in diminutives in Ukrainian. In contrast, the gender assignment of inanimate nouns is arbitrary and unrelated to semantic meaning or biological sex (Corbett, 1991). Because of the arbitrariness of its application across languages and its detachment from conceptual‐ontological meaning, grammatical gender is particularly relevant for discussions of linguistic relativity, as it exemplifies linguistic phenomena that are independent of real‐world differences and are purely linguistic in nature (Bassetti, 2007).

The reason for including Ukrainian‐Russian simultaneous bilinguals stems from the type of language pairing they provide and how this pairing can deepen our understanding of gender effects on cognition. Previously, studies on sequential bilinguals have explored various pairings of languages, such as speakers of a gendered L1 and a genderless L2 (Sato & Athanasopoulos, 2018; Pavlidou & Alvanoudi, 2013; see full discussion in Chen & Faitaki, 2024), a genderless L1 and a gendered L2 (Kurinski & Sera, 2011; Athanasopoulos & Boutonnet, 2016), or a gendered L1 and gendered L2 (Lambelet et al., 2016). Each pairing allows to test for the variability of grammatical gender effects in bilinguals and, in the case of gendered L1‐L2 pairings, to examine the interactions of two grammatical gender structures. Yet, simultaneous bilinguals with two gendered first languages (gendered L1 and 2L1) remain largely unexamined, with only two studies available to our knowledge (Bassetti, 2007; Osypenko et al., 2025). For instance, in the study by Bassetti (2007), Italian‐German bilingual and Italian monolingual children were tested in the voice attribution task (i.e., participants assigning either a male or a female voice to gendered objects). All objects were selected so that their grammatical genders in Italian and German were mismatching (e.g., an object being masculine in Italian and feminine in German, and vice versa). The findings indicated that only the monolingual group exhibited grammatical gender effects on their responses, with Italian monolinguals assigning more male voice to nouns that are masculine in Italian, and a female voice to feminine nouns. Italian‐German bilinguals did not show any effects of either Italian or German grammatical gender on their responses, suggesting that gender mismatch across languages might have reduced grammatical gender effects. Importantly, the study did not examine the effects of grammatical gender for objects whose gender matched across both L1s. Therefore, it remains unclear whether the absence of predicted gender effects was specific to the set of mismatched stimuli, due to gender conflict between the two languages, or whether such effects are generally absent in the chosen participant group as a result of having two gendered L1s.

To address this, Osypenko et al. (2025) studied the effects of having two gendered L1s (Ukrainian and Russian, as in the current study), by investigating both matching and mismatching gendered objects across the two languages, using a similarity judgment task (adapted from Phillips & Boroditsky, 2003). Ukrainian‐Russian adult simultaneous bilinguals with English as an L2 were presented with pairs consisting of a depicted object and a gendered character (a male or a female cartoon character) and asked to rate how similar they are on a Likert scale from 1 (not similar at all) to 9 (very similar). The study had two experiments with the same task and experimental conditions; however, Experiment 1 included stimuli of all three genders represented in Ukrainian and Russian (i.e., masculine, feminine, and neuter), while Experiment 2 excluded those with neuter gender. First, stimuli that were matching in grammatical gender (e.g., “a fork” – feminine in both Ukrainian and Russian) paired with a male/female character resulted in two conditions: congruent/incongruent in both L1s. The prediction was that when pairs were congruent in both L1s, participants would rate them as more similar, compared to the incongruent pairs. Second, stimuli with mismatching grammatical across the L1s (“a notebook” – masculine in Ukrainian, feminine in Russian) paired with male/female characters created conditions where pairs were either congruent in Ukrainian or congruent in Russian. Osypenko et al. (2025) predicted that participants would rate those pairs as more similar that are congruent in their more proficient L1. While Experiment 1 did not reveal the predicted effects of grammatical gender for either type of stimulus, Experiment 2 found these effects for both stimulus types. Specifically, simultaneous bilinguals rated pairs as more similar when the grammatical gender was congruent across both L1s, and when congruency aligned with their more proficient L1. Although alternative explanations for the discrepancy between the two experiments are considered, the findings overall suggest that grammatical gender can influence simultaneous bilinguals of two gendered languages. However, the manifestation of these effects appears to depend on the experimental context, that is, when neuter gender is excluded.

### 1.2 Grammatical gender effects in two‐ versus three‐gendered languages

An additional factor motivating the current study is the ongoing debate over whether grammatical gender effects are present in speakers of three‐gendered languages (i.e., languages with masculine, feminine, and neuter genders) or whether such effects are limited to speakers of two‐gendered languages (i.e., those with only masculine and feminine genders). This discussion emerged in the early 2000s (Sera et al., 2002; Vigliocco et al., 2005) and has continued in more recent work (Osypenko et al., 2025). For instance, Sera et al. (2002) research on German‐English bilingual, as well as French and Spanish monolingual children, revealed that, unlike their monolingual counterparts, German‐English bilinguals did not use German grammatical gender as a basis for assigning voices to objects in a voice attribution task. The researchers speculated that the lack of effects could be attributed to the presence of neuter gender in German, suggesting that languages with a two‐gender system have a strong association between grammatical and natural gender. The latter, according to Sera et al. (2002), leads to overgeneralization of masculine and feminine traits to inanimate objects. In contrast, speakers of languages with a three‐gender system, such as German, appear to rely less on gender and more on other conceptual distinctions (artificial or natural entities) when categorizing objects. Subsequently, Vigliocco et al. (2005) reached a similar conclusion, finding that grammatical gender effects are limited to two‐gendered languages, as evidenced by comparative responses of Italian and German participants. The authors claimed that two‐gendered systems have a high degree of transparent correspondence between the grammatical gender of nouns denoting humans and the biological sex of those humans.

This discussion has evolved with the emergence of evidence either fully (Beller et al., 2015; Bender et al.,^2016^; Haertle, 2017; Maciuszek et al., 2019) or partially (Pavlidou & Alvanoudi, 2013; Osypenko et al., 2025) supporting the presence of grammatical gender effects in speakers of three‐gendered languages, raising further questions about what factors (e.g., experimental design, language typology, etc.) contribute to the discrepancies observed in findings across studies involving speakers of these languages. This inconsistency with earlier findings may be attributed to typological differences between the languages examined. Specifically, the nature and transparency of grammatical gender systems‐including how extensively grammatical gender is marked and the degree of interplay between cultural or conceptual associations and grammatical gender‐varies across language families such as Germanic, Romance, and Slavic (see Kupisch, Geiss, Mitrofanova, & Westergaard, 2022 for further discussion on cross‐linguistic gender transparency). These differences may influence the strength or presence of observed effects, rather than being reduced simply to whether a language has two or three grammatical genders. For instance, unlike Romance languages, German does not have a strong consistency in how grammatical gender of the nouns is referring to humans and their biological sex (e.g., “*das Mädchen*,” translates as “the girl,” yet has neuter gender assigned to it), while in Polish, Ukrainian, and Russian languages, animate entities are referred to either with a masculine or a feminine gender, except for diminutive forms for animals in Ukrainian language (Gorpynyč, 2004). Furthermore, German articles in certain cases do not differentiate between genders. For instance, in the dative case, both masculine and neuter genders would require the same article “*dem*” (e.g., “*der Mann* ” (the man) – “*dem Mann* ” (to the man), “*das Kind* ” (the child) – “*dem Kind* ” (to the child). These factors might have led to less pronounced effects of grammatical gender effects in German speakers, compared to speakers of two‐gendered languages, where grammatical and semantic gender are more closely aligned (Kousta et al., 2008). Slavic languages (e.g., Polish, Ukrainian, Russian) do not have the aforementioned features, as they do not contain articles. Instead, grammatical gender is primarily indicated by the endings of nouns, adjectives, and, in certain cases, verbs. Therefore, including three‐gendered languages free from the constraints mentioned above can lay out a good testing ground to determine whether language effects are indeed solely confined to two‐gendered languages.

A few studies provided evidence in favor of gender effects with a Slavic three‐gendered language, Polish, albeit with monolingual speakers (Haertlé, 2017; Maciuszek et al., 2019). Haertlé’s (2017) study consisted of voice attribution and adjective assignment tasks, conducted in participants’ L1, for 19 objects with mismatching grammatical gender in Polish and French (e.g., “a house” – masculine in Polish, feminine in French). Participants were French and Polish native speakers. Nouns that have neuter gender in Polish were not included in the stimuli. The findings showed significant interactions between language and grammatical gender in a voice attribution task for both Polish and French speakers, with stronger effects in French, suggesting that grammatical gender influences cognitive processes in three‐gendered languages, though the effects were more pronounced in two‐gendered French. However, it is unclear whether these effects would vary if neuter gender stimuli were included. A subsequent study investigating grammatical gender effects in Polish speakers used three different experimental designs: triadic similarity judgments, an implicit association test, and a voice attribution task (Maciuszek et al., 2019). While the triadic similarity judgment task did not show effects of grammatical gender, the other two tasks did. The study highlights that grammatical gender in Polish influences cognitive processes beyond simple categorization, affecting implicit cognition and the attribution of characteristics to objects. Similar to Haertlé (2017), these findings suggest that while two‐gendered languages might exhibit stronger grammatical gender effects, three‐gendered languages like Polish still show significant influences on cognition. Therefore, one can argue that the distinction between two‐ and three‐gendered languages does not determine the lack or presence of language effects; instead, the conditions under which grammatical gender effects emerge warrant closer examination. In line with this conclusion, Osypenko et al. (2025) report supporting evidence. Although Experiment 1 did not reveal the predicted grammatical gender effects in speakers of two three‐gendered languages, Experiment 2 provided evidence for such effects once the neuter gender was excluded from the experiment. This suggests that the presence or absence of neuter gender may influence whether effects are observed. Nonetheless, the study demonstrates that grammatical gender effects on categorization can still be elicited in speakers of three‐gendered languages, even when tested in a genderless second language (English, in the case of Osypenko et al., 2025).

Finally, a crucial aspect of all previously discussed studies is that all of them examined effects of grammatical gender on one cognitive process – categorization. Therefore, the current study adds to this discussion by exploring whether such a grammatical property as grammatical gender plays a role in more complex cognitive functions, such as memory and objects’ mental representations.

### 1.3 Memory recall effects in LR research

Studies examining how cross‐linguistic structural and/or labeling differences impact recall also present contrasting evidence, both in favor (Boroditsky & Schmidt, 2000; Fausey & Boroditsky, 2011; Kirjavainen, Kite, & Piasecki, 2020; Roberson, Davies, & Davidoff, 2000; Tosun, Vaid, & Geraci, 2013) and against Whorfian effects (Cibelli, Xu, Austerweil, Griffiths, & Regier, 2016; Regier & Xu, 2017; Sakarias & Flecken, 2019; Ünal, Pinto, Bunger, & Papafragou, 2016). For instance, Fausey and Boroditsky (2011) investigated how linguistic differences in describing videos of intentional and accidental events (a person pops a balloon using a tack vs. a person reaches to put a tack in a container and accidentally pops the balloon during reach) influence memory recall for agents in English and Spanish speakers. They found that while both groups described intentional events agentively and remembered agents equally well, differences emerged for accidental events. English speakers used more agentive language when describing accidents (e.g., “She popped the balloon”) and showed better memory for the agents involved in these events. In contrast, Spanish speakers, who often used nonagentive constructions (e.g., “The balloon popped”), were less likely to recall the agent responsible for accidental actions.

In a more recent study, examining effects of cross‐linguistic differences on memory recall in a different domain of grammatical number, Kirjavainen et al. (2020) manipulated the presence/absence of compulsory number marking in monolingual speakers of English and Japanese. Across two experiments, participants viewed photos of either one or two objects/animals for 2 s, after which they answered questions about number information (e.g., “How many lions did you see? 1 or 2?”), along with control questions about other details. In Experiment 2, 20 “guessing” questions were added, referencing photos never shown, to assess whether participants were employing a guessing strategy. The results suggested that English speakers, whose language requires explicit singular/plural marking (e.g., “apple” vs. “apples”), better recalled plurality information. In contrast, Japanese speakers, whose language allows omission of number marking, showed significantly lower accuracy in recalling plural items. Experiment 2 confirmed that this effect was not due to guessing or question wording.

Overall, a large body of research provides support for both lexical and grammatical properties influencing recall. However, existing evidence calls into question whether these findings show a true Whorfian effect (i.e., language affecting perception) or rather “language‐on‐language” effects (i.e., participants use language to complete a language‐engaging task; Wolff & Holmes,^2011^). To address these two possibilities, Sakarias and Flecken (2019) investigated how case markings in Estonian and Dutch influence attention allocation in verbal and nonverbal event encoding and memory recall of the event endings. In the current review, we limit our discussion to the recall‐related findings, as they are most relevant here. Two types of events were chosen for the study: resultative events (where objects undergo a visually noticeable change in state during the event, e.g., peeling a potato) or nonresultative events (no or only partial change of the object's state, e.g., stirring in a pan). The Estonian language has obligatory case marking when objects sustain a partial/full change in state (e.g., a fully peeled potato marked by accusative case “*kartuli*,” whereas a partially peeled potato—by partitive case “*kartulit*”). On the other hand, Dutch lacks such grammatical marking (e.g., “*een aardappel schillen*” can mean both partial and full peeling). Participants watched short video clips depicting everyday causative events and were then required either to verbally describe the videos or to complete a nonverbal distractor task involving detecting auditory cues. Afterward, participants performed a surprise forced‐choice recognition memory task testing their memory for the event endings. Sakarias and Flecken (2019) found a language‐specific boost on recall of event results among Estonian participants compared to Dutch, but only under the verbal task. Specifically, Estonian speakers exhibited superior memory recall for video endings only in the verbal condition. Therefore, the findings were interpreted as supporting not the true Whorfian effect, but rather thinking‐for‐speaking effects (i.e., case marking influenced event memory only within language‐dependent contexts; see Slobin, 1996; Wolff & Holmes, 2011), as no significant language‐specific differences emerged in the nonverbal encoding condition.

As for memory recall studies examining the effects of grammatical gender, the evidence, to date, has been fairly scarce. In their review, Samuel et al. (2019) report that this paradigm comprises only 2% of all studies that were selected to analyze ways researchers can investigate cross‐linguistic linguistic relativity effects of grammatical gender. In total, this task has been employed in three distinct research studies and provided a combination of mixed support and no support (Boroditsky & Schmidt, 2000; Kaushanskaya & Smith, 2016; Pavlidou & Alvanoudi, 2013). Given the centrality of memory in the human cognitive system (Baddeley & Hitch, 1974), and the robust manifestation of linguistic relativity effects on memory in other linguistic domains (Lucy, 1992; Athanasopoulos & Bylund, 2013; Roberson et al., 2005), the current study aims to redress the balance of evidence of possible Whorfian effects on memory recall in the domain of grammatical gender.

The chosen methodological approach was originally developed by Boroditsky and Schmidt (2000). In their study, sequential bilinguals (25 Spanish−English and 16 German−English) and 20 English monolinguals were tasked with memorizing a male/female name placed next to an object possessing a distinct grammatical gender in the participant's native language.For example, “a chair” that is masculine in German (“der Stuhl”) and feminine in Spanish (“la silla”) was paired with either a male name (e.g., Patrick) or with a female name (e.g., Patricia). All objects had opposite genders in Spanish and German. Half of the names had a biological sex that was congruent with the grammatical gender of the paired object in L1, and the other half was incongruent. Participants’ ability to recall these word‐name combinations was then assessed. Boroditsky and Schmidt (2000) found effects of grammatical gender in native speakers of Spanish and German while being tested in English. Specifically, both Spanish‐English and German‐English bilinguals recalled better those name‐object pairs where the biological sex of a proper name was congruent with the grammatical gender of the object in their native language (82% and 74% correct responses respectively, *t* = 2.55, *p* <.01). Therefore, since the objects chosen for the study had opposite grammatical genders in German and Spanish (e.g., feminine in Spanish and masculine in German, and vice versa), participants show opposite memory biases. For objects that Spanish speakers were more likely to remember paired with female names, German participants remembered them when they were paired with male names, and vice versa. While having certain limitations (i.e., effects of conceptual gender, sample size, etc.), the presented conclusion holds significant importance as it suggests that both two‐and three‐gendered languages exert comparable effects on cognition, despite the presence of neutral grammatical gender in German.

In a subsequent iteration, Pavlidou and Alvanoudi (2013) adapted this framework with Greek−English sequential bilingual speakers, Greek being a three‐gendered language. The stimuli comprised 28 nouns, each accompanied by a unique proper Greek name (e.g., Vasilis/Vasiliki, Alekos/Aleka) and then automatically followed by another pair in a randomized order. It was hypothesized that participants’ memory would be more effective when the grammatical gender of words/objects in their L1 matched with the gender of proper names compared to cases where such alignment was absent. However, the authors reported that the memory task did not show any effects of the congruence between the grammatical gender of nouns and the biological sex of the names. Pavlidou and Alvanoudi (2013) attribute the nonreplication of the memory task to methodological variations between their study and the original study by Boroditsky and Schmidt (2000). Although the tasks were similar, Pavlidou and Alvanoudi (2013) were unable to replicate the procedure exactly due to limited detail in the original methodological descriptions, emphasizing the need for greater transparency in methodological design to facilitate replication. Additionally, going back to the discussion on three‐gendered languages, Pavlidou and Alvanoudi (2013) do not speculate whether the lack of results can be explained by the Greek language being three‐gendered.

Finally, Kaushanskaya and Smith (2016) used a similar experimental design as Boroditsky and Schmidt (2000) while looking at the reversed language pairing in their bilingual participant group (genderless English as an L1 and a two‐gendered Spanish as an L2) to examine whether grammatical gender information from a second language could influence memory performance in a first language that lacks gender marking. Three groups of English L1 speakers were tested: monolinguals, emergent bilinguals with high exposure to Spanish, and those with low exposure. Analogously to the previous two studies (Boroditsky & Schmidt, 2000; Pavlidou & Alvanoudi, 2013), participants completed an associative learning task, pairing inanimate object names with gendered proper names. The Spanish translation of each object was either gender‐congruent or gender‐incongruent with the name (e.g., corn‐Patrick vs. beach‐William). Crucially, the task was conducted in English. The results showed that high‐exposure bilinguals exhibited sensitivity to Spanish grammatical gender: they were significantly less accurate in recalling incongruent pairs compared to congruent ones. In contrast, monolinguals and low‐exposure bilinguals showed no such effect. These findings suggest that grammatical gender information from a second language can be activated and influence memory performance, even during tasks conducted entirely in the native, genderless language. In doing so, Kaushanskaya and Smith (2016) provide evidence not only for grammatical gender effects on recall, but also for the potential of L2 grammatical properties to restructure bilinguals’ cognitive processing.

### 1.4 The current study

The present study extends the examination of grammatical gender effects on cognition by employing a memory task adapted for Ukrainian‐Russian simultaneous bilinguals. In addressing our hypotheses and research questions, we refined the methodology and the analysis from Boroditsky and Schmidt's (2000) original study in several ways. First, instead of sequential bilinguals, we recruited simultaneous speakers of Ukrainian and Russian. Second, instead of comparing Spanish−English and German−English bilinguals, where one group has a two‐gendered language and another group has a three‐gendered language, we recruited speakers who have two three‐gendered grammatical systems embedded in their L1s. Third, we expanded the stimuli list from 24 to 46 nouns. Instead of solely relying on stimuli with opposite grammatical genders in German and Spanish for between‐subject comparison, we opted for a dual approach with two types of stimuli. This allowed us to conduct between‐subject analysis in the first part of the study, comparing Ukrainian−Russian bilinguals and English monolinguals, where chosen nouns had matching grammatical gender in both L1s (e.g., “key” – masculine in both Ukrainian and Russian, “strawberry” – feminine in both, “feather” – neuter in both). In the second part, analyzing only the performance of Ukrainian−Russian bilinguals, we employed a within‐participant design to explore mismatching grammatical genders across two L1s (e.g., “moon” – masculine in Ukrainian, feminine in Russian, “sock” – feminine in Ukrainian, masculine in Russian). Lastly, we added a more detailed linguistic profile analysis, as well as proficiency tests, to analyze the effect of language proficiency in bilinguals’ both L1s on their performance.

Building on the previous research, we predicted that the effects of native language(s) on the memory recall of object‐name pairs presented to participants will be observed. Specifically, the recall of the names by Ukrainian−Russian bilinguals is hypothesized to be enhanced when the grammatical gender of the noun is congruent with the biological sex of the name in participants’ native language(s), compared to English monolinguals. The hypotheses were formulated based on the two types of selected stimuli. First, for nouns with matching grammatical gender in both native languages, we expect to find a stronger language effect on the ability to remember the assigned names, compared to English‐speaking controls. For instance, bilingual participants are expected to remember the pair “Patrick – key” better than “Patricia – key,” as “key” is masculine in both Ukrainian and Russian and is congruent with male biological sex. Besides, we anticipate a more pronounced language effect for the stimuli with the matching gender across two languages, compared to the mismatching one, since the converging grammatical information from the two languages would lead to stronger memory associations.

Second, for nouns with mismatching grammatical gender among bilingual participants, we anticipate that participants will display an effect of their more proficient language when recalling the names. For example, if a participant is more proficient in Ukrainian rather than Russian, they will tend to remember those names where gender matches Ukrainian and mismatches Russian. Participants with greater proficiency in Ukrainian than Russian are expected to show higher accuracy when recalling the pair “Eric – moon” compared to “Erica – moon” (and vice versa for those more proficient in Russian), as “moon” carries masculine grammatical gender in Ukrainian and feminine in Russian.

## 2 Methods

The materials, data, and analysis codes for this study can be retrieved from the OSF link: https://osf.io/xhs9v/.

### 2.1 Pre‐test

To exclude conceptual gender from the analysis and focus solely on the effects of grammatical gender, we carried out a pre‐test using the methodology outlined by Sato and Athanasopoulos (2018). This was done to select conceptually neutral items for the main experiment. We recruited 10 Ukrainian−Russian−English speakers (*5 females*; *Mean age = 26*, *SD age = 4*) and 10 English monolinguals (*4 females*; *Mean age = 31*, *SD age = 10*). None of the recruited participants were involved in the main study. Participants were asked to rate 137 black‐and‐white object images presented one by one against a grayscale and white background to minimize any bias related to color. The objects were rated on a Likert scale ranging from “very feminine” (1) to “very masculine” (7). The objects were divided into five groups based on their grammatical genders in Ukrainian and Russian: (1) 20 nouns masculine in Russian and feminine in Ukrainian; (2) 24 nouns feminine in Russian and masculine in Ukrainian; (3) 31 nouns feminine in both languages; (4) 31 nouns masculine in both languages; and (5) 31 nouns neutral in both languages. The images used in the study were obtained from the Bank of Standardized Stimuli (Brodeur, Guérard, & Bouras, 2014) and the Snodgrass and Vanderwart (1980) database.

### 2.2 Stimuli

From the pre‐test, we obtained 46 conceptually neutral objects (*Mean = 4.04; SD = 0.07; Range = 3.85–4.1*). These objects were then divided into three groups (Table 1): (1) 18 objects with matching grammatical gender in both Russian and Ukrainian (e.g., blender—masculine in both); (2) 20 objects with mismatching grammatical genders (e.g., tray—feminine in Ukrainian, masculine in Russian); and (3) eight filler objects with neuter grammatical gender in both languages. It is important to point out that given the typological proximity and lexical overlap between Ukrainian and Russian, the presence of cognates in the stimulus set was largely unavoidable. While the pre‐test included a broader mix of cognates and noncognates, the final selection was determined exclusively based on conceptual gender neutrality, which resulted in an uneven distribution of cognates across conditions. Specifically, 89% (23 out of 26, including fillers) of nouns in the matched‐gender group were cognates (e.g., “cutting board” ‐“*дошка* ” in Ukrainian and “*доска* ” in Russian; “guitar” ‐“*гітара* ” in Ukrainian and “*гитара*” in Russian), whereas only one noun (5%) in the mismatched‐gender group was a partial cognate (e.g., “parrot” ‐“*папуга* ” in Ukrainian and “*попугай* ” in Russian). Although cognate status was not systematically manipulated in this study, we recognize that it may have influenced bilingual lexical processing.

**Table 1:** Example of the types of stimuli used for the current study

Types of stimuli	Ukrainian grammatical gender	Russian grammatical gender	Number of nouns	Example (English/Ukrainian/Russian)	English word length	Ukrainian word length	Russian word length
Matching grammatical gender across both L1s	Masculine		9	Wineglass/Келих/Бокал Tomato/Помідор/Помидор	Mean = 6.78, SD = 2.60 Range = 3−13	Mean = 6.72, SD = 2.30 Range = 4−13	Mean = 7.11 SD = 3.20 Range = 4−17
	Feminine		9	Box/Коробка/Коробка Candle/Свічка/Свеча			
Mismatching grammatical gender across both L1s	Masculine	Feminine	9	Basket/Кошик/Корзина Notebook/Зошит/Тетрадь	Mean = 7.05, SD = 3.69 Range = 3−16	Mean = 7.35, SD = 3.31 Range = 4−15	Mean = 7.10 SD = 3.55 Range = 3−16
	Feminine	Masculine	11	Umbrella/Парасолька/Зонт Onion/Цибуля/Лук			
Fillers	Neuter		8	Apple/Яблоко/Яблуко Feather/Перо/Перо	Mean = 5.12, SD = 1.25 Range = 3−7	Mean = 5.12, SD = 1.13 Range = 4−7	Mean = 4.62 SD = 1.06 Range = 3−6

*Note*. All stimuli were presented in pictorial format from established image databases.

We also acknowledge the slight imbalance between the two groups of stimuli (Table 1). Given the constraints in selecting conceptually neutral objects with mismatching grammatical gender in both Russian and Ukrainian, we prioritized internal balance within each analyzed category. Specifically, the matching grammatical gender group was designed to be balanced (nine masculine, nine feminine), while the mismatching group was constructed with the most conceptually neutral items available, which resulted in a slight difference in total count. Importantly, in the mismatching group for the variable “Condition” (i.e., Congruent in Russian/Incongruent in Ukrainian vs. Congruent in Ukrainian/Incongruent in Russian), we examined the effects of participants’ most proficient L1 (Ukrainian or Russian) across all mismatching nouns (9 masculine‐feminine and 11 feminine‐masculine) combined. Therefore, the slight numerical imbalance is not expected to affect the interpretation of our results. Finally, descriptive statistics for word length across all three languages are provided in Table 1. These show that word lengths are comparable across stimulus types and languages.

Each of the nouns was paired up with either a male or female name, with names counterbalanced per participant. The names in the study were retained from the original study (see Table 2) due to the limited availability of names with a comparable number of syllables in both the Ukrainian and Russian languages.

**Table 2:** Names used in the study by Schmidt and Boroditsky (2000)

*Male names*	*Female names*
Christopher	Christina
Daniel	Danielle
Paul	Paula
Brandon	Brenda
Eric	Erica
Karl	Karla
Claude	Claudia
Phillip	Phyllis
Harry	Harriet
Donald	Donna
Alexander	Alexandra
Patrick	Patricia

Overall, for the current experiment, four experimental conditions were established based on the two types of stimuli. For the stimuli with matching grammatical gender, two conditions were delineated based on the congruence between the biological sex of the name and the noun's grammatical gender in bilinguals’ both L1s: (1) Congruent in both L1s and (2) Incongruent in both L1s. Analogously, for the second type of stimuli (with mismatching grammatical gender), we defined two conditions: (1) Congruent in Ukrainian & Incongruent in Russian and (2) Congruent in Russian & Incongruent in Ukrainian.

### 2.3 Participants

We recruited 100 Ukrainian−Russian bilinguals and 40 English monolinguals in exchange for an inconvenience allowance of £10 in the form of an Amazon voucher. After analyzing the responses and linguistic profiles of the participants, our final sample consisted of 94 Ukrainian participants (*70 females*, *Mean age = 32*, *SD age = 12.1*) and 38 English monolinguals (*21 females*, *Mean age = 23*, *SD age = 2.5*). Participants were removed for reasons such as speaking/learning another gendered language (*n = 7*) or showing unusually slow reaction times between stimuli (*n = 1*). The imbalance between male and female bilinguals was due to data collection occurring after the start of the war in Ukraine, resulting in a skewness of the available sample. Nevertheless, Flaherty (2001) reported, based on statistical analysis in a sex assignment task, that while the sex of participants influenced the responses in the younger group (5‐ to 7‐year‐olds and 8‐ to 10‐year‐olds for Spanish participants, and 5‐ to 7‐year‐olds for English participants), both for Spanish and English adults, the sex of the participants did not affect the choices of male or female gender for the nouns (*χ* ^2^ *= .8606*, *ns*, *and χ* ^2^ *= 2.88*, *ns*, *for Spanish and English adults*, *respectively*).

Proficiency levels in Ukrainian, Russian, and English for Ukrainian participants were gauged through standardized tests. The ZNO Tests (Ukrainian Center for Educational Quality Assessment, 2020) were used to evaluate advanced language skills in Ukrainian and Russian (on a scale from C1 to C2 levels). Participants could score a maximum of 100 points for each language. To calculate a continuous variable for language proficiency, scores from the Russian proficiency test were subtracted from those of Ukrainian. Consequently, this coefficient could range from a maximum of +100, indicating exclusive proficiency in Ukrainian, to a minimum of −100, signifying exclusive proficiency in Russian. English language proficiency was measured using the Oxford Quick Placement test, OQTP (Oxford University Press, 2001), or by evaluating existing valid IELTS scores (Cambridge University Press, 2021). The minimum acceptable scores were set at 67% for the OQPT and an IELTS score of 5.5, corresponding to a B2 (upper‐intermediate) proficiency level. Both groups also completed a modified Bilingual Language Profile questionnaire (BLP, Gertken et al., 2014) to determine if they spoke any other languages.

Ukrainian participants included in the analysis reported acquiring English as a foreign language at an average age of 10 (*SD = 4.21*) with a minimum of upper‐intermediate proficiency level. Most participants showed the highest proficiency scores in Ukrainian (*55%*), followed by Russian (*27%*), and equal proficiency in both languages (*18%*). The proficiency scores also varied greatly (Range _Ukrainian_ = 6.25−93.75, Range _Russian_ = 6.25−87.5, with 100 being a maximum score), indicating the absence of ceiling effects (see Table 3 for more details).

**Table 3:** Proficiency scores and distribution of Ukrainian−Russian bilingual participants

Language	Mean proficiency score (100 maximum)	SD	Range	Percentage (number) of participants
Ukrainian	66.69	14.96	6.25−93.75	55% (52)
Russian	59.11	14.45	6.25–87.5	27% (25)
Equal proficiency in both	62.13	14.03	31.25−87.5	18% (17)

All participants were recruited either online or via posters distributed at Lancaster University. The Research Ethics Committee at Lancaster University approved the study protocol and the data collection measures.

### 2.4 Procedure

To conduct the current experiment, we used the Gorilla Experiment Builder software (Anwyl‐Irvine, Massonnié, Flitton, Kirkham, & Evershed, 2019). Participants were monitored online to ensure the integrity of their performance on the memory tasks.

The experiment was conducted in English and consisted of two phases: learning and testing, repeated twice. Following the study by Boroditsky and Schmidt (2000), participants were presented with the following instructions: “*For this experiment*, *we have given names to a bunch of objects. For example*, *we may have decided to call a chair ‘Mary’. You will see objects and their names appear on the screen (e.g*., *chair Mary)*, *and your task is to try to memorize the name we have given to each object as well as you can. Your memory for these names will be tested later in the experiment*.”

**Fig. 1:**
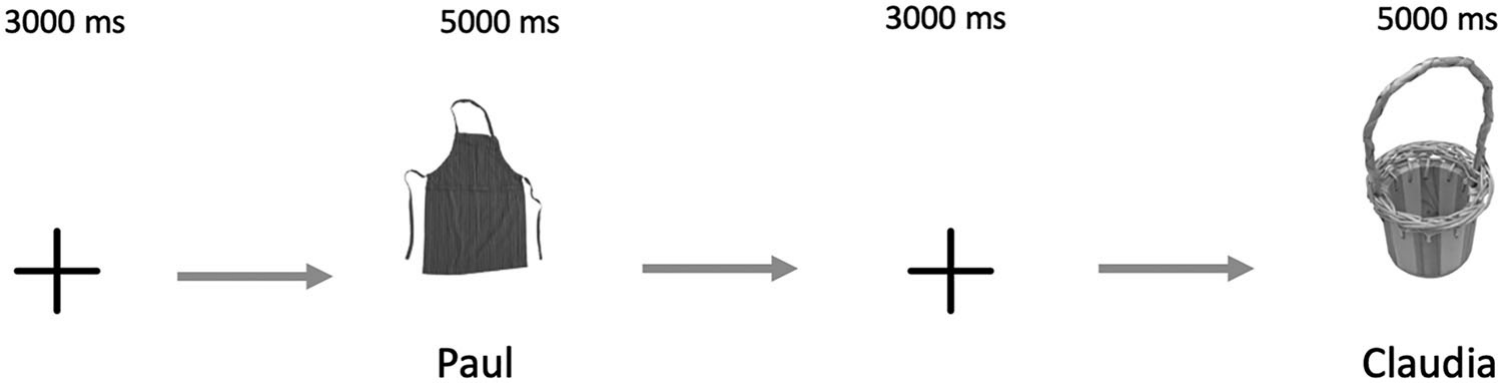
Example of the stimuli used in the learning phase.

Then, participants were presented with 23 object–name pairs. The pairs appeared on the screen for 5 s each, with the object in black‐and‐white presented in the center of the screen and the name displayed below (Fig. 1). Each object was shown only once per participant. Crucially, the gender of the name associated with each depicted noun was counterbalanced across participants. For instance, one participant might view “apple” paired with the name “Patrick,” whereas another was presented the same object paired with “Patricia.” This between‐subjects counterbalancing ensured that each object was paired with both a masculine and a feminine name across the sample, but never more than once per participant. As such, there were no within‐subject repetitions of objects, thereby minimizing potential carryover effects from earlier exposures.

**Fig. 2:**
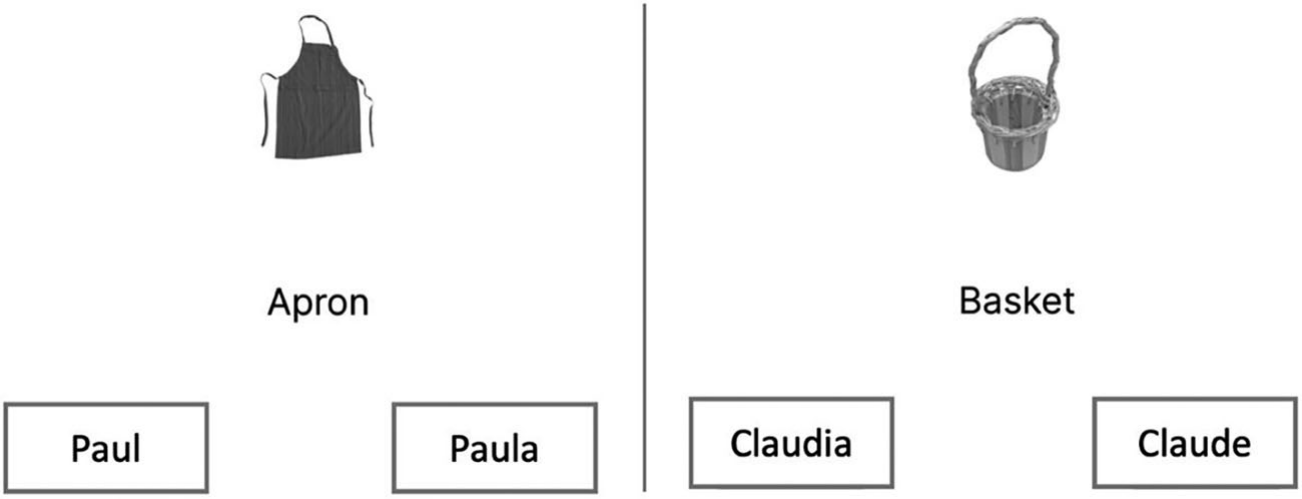
Example of the stimuli used in the testing phase.

Afterward, participants completed an unrelated distractor task, which typically lasted 2−3 min. Since the original study did not specify the distractor task, we included a Thatcher task and a semantic priming task. During the testing phase, object names were shown on the screen, and participants had to select the gender of the proper name from the learning phase (e.g., choosing between “Daniel” and “Danielle,” see Fig. 2). Since our study had twice the number of stimuli as in the original study, all participants repeated this process twice (23 pairs per trial). Each of the names was repeated only once per trial. After the second session, participants completed language proficiency tests and a BLP questionnaire.

### 2.5 Analysis

Considering the intricacies of our study, we divided the analysis into two parts, based on the two types of stimuli. The first part focused on a comparative analysis of responses from Ukrainian−Russian bilingual and English monolingual participants, using the group of nouns that had matching grammatical gender in Ukrainian and Russian. In this part of the analysis, our aim was to replicate the findings of Boroditsky and Schmidt (2000), providing evidence that the ability to recall the human name by simultaneous bilinguals is enhanced if its biological sex is congruent with the grammatical gender of the object in question in both L1 and 2L1, compared to the English monolinguals. To achieve this, we designed a generalized linear mixed‐effects (lmer) model (Linck & Cunnings, 2015) in R software (R Core Team, 2022) to determine whether the accuracy of responses (correct vs. wrong, coded as 1 and 0) was influenced by condition (Congruent in both L1s vs. Incongruent in both L1s, contrast coded as 0.5 and −0.5) and the participant group (bilingual vs. monolingual, contrast coded as 0.5 and −0.5). The parsimonious model included by‐participant random intercepts and slopes for condition, and by‐item random intercepts. Additionally, we analyzed whether there was a difference in the recall accuracy within the Ukrainian−Russian bilingual group depending on the condition. For that, we designed an lmer model with accuracy (“1” for accurate and “0” for inaccurate responses) as a dependent variable, and the condition as a predictor. The maximal model included random intercepts and slopes for condition both by participant and item. However, due to a singular fit and near‐zero variance for participant‐level random effects, the parsimonious model retained only random intercepts for items.

For the second part of the analysis, looking at the group of nouns where grammatical gender was mismatching across languages, we explore further the effects of two contrasting three‐gendered systems on memory and whether more proficient L1 (Ukrainian or Russian) will affect the accuracy of the responses, compared to the less proficient L1. To do this, we analyzed how the accuracy of responses is affected by the interaction between Proficiency (measured from −100 to +100 for Russian and Ukrainian, respectively) and Condition (Congruent in Russian & Incongruent in Ukrainian vs. Congruent in Ukrainian & Incongruent in Russian, contrast coded as −0.5 and 0.5). The parsimonious model for this analysis also included a random intercept for Item to account for variability across stimuli. Participant‐level variability was not included due to zero variance and convergence issues in the maximal model.

## 3 Results

### 3.1 Comparison of English monolingual and Ukrainian−Russian bilingual participants

**Fig. 3:**
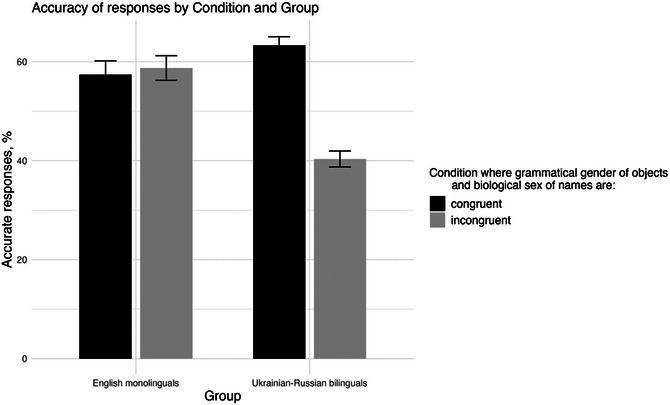
Accuracy of responses (%) based on condition (Congruent in both L1s and Incongruent in both L1s) by group (English monolinguals and Ukrainian−Russian bilinguals). Error bars indicate 95% confidence intervals.

Aligning with our predictions, bilingual participants recalled names more accurately when the grammatical gender of objects in both Ukrainian and Russian languages aligned with the biological sex of the names assigned to them during the learning phase (*Mean = 63%*, *SE = 1.75*). This was compared to the accuracy of responses for objects whose grammatical gender in both languages misaligned with the biological sex of the names (*Mean = 40%*, *SE = 1.61*), as well as comparing with the responses of English monolinguals in both conditions (Fig. 3). As expected, the English control group did not display any significant trends, regardless of whether the grammatical gender and biological sex of the object names were congruent (*Mean = 57%*, *SE = 2.8*) or incongruent (*Mean = 58.7%*, *SE = 2.5*) in Ukrainian and Russian. This suggests that congruency between the grammatical gender and biological sex of the names improves recall accuracy in Ukrainian‐Russian bilinguals, compared to monolingual participants, supporting our hypothesis.

In the linear mixed model, the dependent variable (Accuracy:correct vs.wrong response) was significantly affected by both main effects of participant group *(Estimate =* −*0.247*, *SE = 0.010*, *z =* −*2.48*, *p = .013)* and the condition *(Estimate =* −*0.687*, *SE = 0.277*, *z =* −*2.48*, *p = .013)*. As predicted, the interaction between group and condition was also significant (*Estimate =* −*1.231*, *SE = 0.377*, *z =* −*3.27*, *p = .001*). Additionally, the comparison of the models with and without the interaction between the predictors revealed a significant improvement in fit when the interaction term between the two variables was included (*χ* ^2^ *(1)= 10.40*, *p = .001*).

When comparing the accuracy of responses for the two conditions (Congruent in both L1s vs. Incongruent in both L1s) only withing a Ukrainian‐Russian bilingual group, we also found a significant effect of the condition *(Estimate =* −*1.080*, *SE = 0.244*, *z =* −*4.44*, *p <.001)*, suggesting that bilinguals recalled significantly less accurate those pairs in the “Incongruent in both L1s” condition.

### 3.2 Comparison of Ukrainian−Russian bilingual participants based on language proficiency

For the second part of the analysis, we investigated whether bilingual participants would show improved recall of object names when those objects were paired with items whose grammatical gender in their more proficient language (Ukrainian or Russian) was congruent with the biological sex of the name. This was compared to the recall of names where the biological sex of the names was congruent with the grammatical gender of the paired object in the less proficient language.

However, no significant effects were found for the main effects of group proficiency *(Estimate* = −*0.0003*, *SE = 0.003*, *z* = −*0.08*, *p = .935)*, condition *(Estimate =* −*0.032*, *SE = 0.482*, *z =* −*0.07*, *p = .948)*, or group proficiency‐condition interaction *(Estimate =* −*0.006*, *SE = 0.006*, *z =* −*0.93*, *p = .352)*. Moreover, when comparing the two models using ANOVA, we found that the model including the two predictors did not provide a significantly better fit (*χ* ^2^ *(1)= 0.00*, *p = 1.00*) compared to the null model.

## 4 Discussion and conclusion

In the current study, our aim was to provide a deeper understanding of language effects on the mental representation of objects, investigate the effects of three‐gendered languages, as well as introduce participants who have two three‐gendered grammatical systems acquired simultaneously. The latter would allow us to provide deeper insights into how such languages interact in a bilingual mind, as well as the effects they have on human cognition.

In the first part of our analysis, comparing the performance of Ukrainian‐Russian bilingual and English monolingual groups, we confirmed our hypothesis. Particularly, we found that during the testing phase, the ability of bilingual participants to recall the names assigned to objects in the learning phase significantly improved when the grammatical gender of objects and the biological sex of the names were congruent in both of their native languages. In this part of the analysis, our results aligned with our initial predictions, notwithstanding the inclusion of neutral stimuli as fillers and our attempt to minimize conceptual relatedness by exclusively including conceptually neutral objects.

However, when analyzing the results of the bilingual group using stimuli where objects had mismatching grammatical genders in Ukrainian and Russian, no effect of the more proficient language was found. This suggests that in the case of simultaneous bilinguals, the effects of language may be negated when there is a misalignment in grammatical gender between the L1 and 2L1. Nevertheless, despite not finding effects for the second type of stimuli, we were able to replicate the original study (Boroditsky & Schmidt, 2000) and find similar results in simultaneous bilingual participants as those of sequential bilinguals, despite both L1 and 2L1 being three‐gendered.

The discrepancy in our findings with the first and second types of stimuli, as well as with earlier studies indicating no effect of three‐gendered languages (Sera et al., 2002; Vigliocco et al., 2005), can be explained in various ways. First, as mentioned before, previous studies mainly compared speakers of two‐ and three‐gendered languages with each other or with a monolingual control group. However, none of these studies investigated the potential language effects that arise when individuals acquire two three‐gendered languages simultaneously. Our findings aligning with the first prediction can be attributed to the chosen first group of stimuli that had matching grammatical gender in Ukrainian and Russian. Therefore, there was no conflict or interference between the gender representations, as opposed to the second type with mismatching genders. Second, when interpreted through the lens of Baddeley's Working Memory Model (Baddeley & Hitch, 1974), which suggests that the central executive, phonological loop, and visuospatial sketchpad work together to process and integrate information, the enhanced recall accuracy observed in bilingual participants when grammatical gender and biological sex were congruent across both L1s suggests that converging grammatical information created stronger memory associations. This likely reduced the cognitive load on the central executive, allowing for more efficient retrieval. However, when grammatical gender and biological sex were congruent in one language and incongruent in another, the increased cognitive load may have increased, leading to weaker memory associations and lower recall accuracy. This is particularly supported by the fact that memory recall for these items was worse than that of the English monolingual controls (see Fig. 3). However, an alternative explanation^1^ is that in cases where bilingual participants were unsure of the correct answer, their guessing behavior—whether conscious or unconscious—may have been influenced by the grammatical gender of the object presented on screen during the recall phase. In pairs where the grammatical gender of the noun was matching across Ukrainian and Russian, this incidental influence could have increased the likelihood of a correct guess, inflating accuracy relative to English monolinguals, who lack such grammatical associations. Importantly, this account is also supported by the lack of a significant reaction time difference (see Supplementary Materials) between bilingual Ukrainian‐Russian and monolingual English participants. Specifically, if bilinguals were benefitting from more efficient memory retrieval due to gender congruency, we might expect faster reaction times in those trials. However, the absence of a reaction time advantage suggests that their improved accuracy may not stem from faster recall, but rather from a bias in guessing behavior. Additionally, in pairs where grammatical gender mismatched across L1s, such a guessing (conscious or unconscious) strategy was not accessible, as the presented objects activated both masculine and feminine gender information.

Another possibility to explain our findings, specifically the discrepancy between stimuli with matching and mismatching genders across two L1s, as suggested by an anonymous reviewer, is that the observed accuracy advantage for congruent pairs using nouns with matching gender in Ukrainian and Russian among bilingual participants may have arisen not from enhanced memory retrieval per se, but from an unconscious (or even strategic) influence of grammatical gender as a result of guessing. In other words, when bilinguals were uncertain of the correct name, they may have defaulted, consciously or unconsciously, to choosing the gendered name that was congruent with the grammatical genders across both L1s of the object presented on screen. Whereas for stimuli with mismatching grammatical genders in Ukrainian and Russian, this strategy was unavailable, as the object might have activated both masculine and feminine gender information, making guessing a more difficult task. Given the number of stimuli per trial (*n* = 23), such guessing strategies cannot be ruled out entirely. However, several aspects of our data suggest that while it could be an unconscious effect of grammatical gender, it is unlikely to be a conscious strategy or guessing.

First, none of the participants mentioned grammatical gender or language as a tool they used during the task in post‐experimental debriefing. Instead, they referred to associations or visual mnemonic strategies, suggesting that any influence of grammatical gender likely occurred implicitly. Second, as mentioned above, our reaction time data revealed no significant differences between bilingual and English monolingual participants, even in trials involving congruent items. In a separate model, we also compared the response times for stimuli with matching grammatical genders across Ukrainian and Russian (conditions “Congruent in both L1s” and “Incongruent in both L1s”) only for Ukrainian−Russian bilinguals and no significant differences were observed. If participants had relied on grammatical gender as a conscious cue during guessing, we would expect to see faster responses for the “Congruent in both L1s” condition, particularly among the bilingual group only—yet, no such pattern emerged. The absence of such effects further supports the idea that grammatical gender influenced recall at an implicit, conceptual level, rather than through deliberate response strategies. Nonetheless, we acknowledge that it is not possible to fully rule out that our findings reflect a conscious strategy or a guess in behavioral experiments (see Samuel et al., 2019). To fully separate conscious language manifestation from unconscious pre‐linguistic Whorfian effects, future studies could incorporate neural measures (e.g., electroencephalography) and go beyond behavioral findings that are commonly facing such critique.

In addition, previous studies comparing the results of bilingual speakers fail to mention whether the presented stimuli were cognates or noncognates. As detailed in Section 2.2, the majority of nouns with matching grammatical gender across Ukrainian and Russian were cognates, whereas cognates were largely absent in the mismatched‐gender condition. This asymmetry may have contributed to the observed differences in recall performance between conditions. While studies in linguistic relativity have not addressed the influence of cognates on grammatical gender effects in bilingual speakers, prior research on bilingual language processing shows a clear advantage of cognates when comparing the speed of translation of words by bilinguals (Degroot, Dannenburg, & Vanhell, 1994; Kroll & Stewart, 1994; Sánches‐Casas, García‐Albea, & Davis, 1992; Salamoura & Williams, 2007). Therefore, we propose that the presence of cognates may have contributed to the enhancement of the language effects found in the recall of names in pairs where nouns had matching grammatical gender, compared to the mismatching ones. The latter also fits into the predictions based on the Working Memory Model (Baddeley & Hitch, 1974), specifically, because cognates facilitate the retrieval of the top‐down information even more when participants categorize the stimuli.

Moreover, our findings are consistent with studies examining Polish grammatical gender (Haertlé, 2017; Maciuszek et al., 2019). This raises the question: what do these languages—Polish, Ukrainian, and Russian—have in common that sets them apart from other three‐gendered languages such as Greek or German? All three are Slavic languages, with Ukrainian and Russian belonging to the East Slavic branch, and Polish to the West Slavic branch. Ukrainian and Polish have the shortest lexical distance at 30%, followed by Ukrainian and Russian at 38%, and Russian and Polish at 50% (Steinback, 2015). They also do not have articles, and grammatical gender is inferred from the noun itself, contrary to Greek and German. In all three languages, verbs in the past tense agree with the gender of the subjects, which is not present in many Indo‐European languages, including Greek and German. For instance, in Russian, “he went” is “он пошёл” (on poshol), “she went” is “она пошла” (ona poshla), and “it went” (for neuter) is “оно пошло” (ono poshlo). This broad grammatical distribution of gender marking may increase its salience and facilitate top‐down processing in memory tasks, as gender is marked on prominent grammatical constituents like nouns and verbs rather than modifiers.

While Ukrainian and Russian are less gender transparent than Romance languages such as Spanish—which occupies the high end of the gender transparency continuum (Kupisch et al., 2022; Sá‐Leite & Lago, 2024) and have been suggested to elicit stronger grammatical gender effects in earlier linguistic relativity studies (Sera et al., 2002)—they are more transparent than German. The latter has been argued to produce weaker gender effects in prior studies (e.g., Sera et al., 2002; Vigliocco et al., 2005), possibly due to its high opacity and the presence of neuter gender, which reduces alignment between grammatical and natural gender.

Crucially, these differences may affect how grammatical gender information becomes integrated into conceptual memory. According to the gender adaptation of the AUSTRAL model (Sá‐Leite & Lago, 2024; see Taft, 2023 for the original AUSTRAL model), gender can be accessed both via form‐based (e.g., consistent morphological endings) and lemma‐based (e.g., repeated and syntactically distributed agreement patterns) routes. However, lemma‐level activation in Ukrainian and Russian may place higher cognitive demands on the language user, as gender information must be maintained and retrieved across multiple syntactic constituents—and in the absence of overt morphological cues like determiners (as in German or Spanish). Ukrainian and Russian are more transparent than German in terms of grammatical gender marking, meaning that the form‐based (or sub‐lexical) route is more frequently used alongside the lexical route to activate and retrieve gender information. This higher transparency results in greater processing load, which may enhance encoding into memory.^2^ In contrast, while German also stores gender at the lemma level, its more limited grammatical embedding of gender (e.g., use of invariable determiners) may lead to weaker conceptual integration. This interpretation aligns with broader findings that higher cognitive, or memory load, tends to increase reliance on language as a resource (e.g., Winawer et al., 2007; Bylund & Athanasopoulos, 2017. Finally, future research could examine this proposal more directly through cross‐linguistic studies comparing languages of varying transparency, and by isolating regular versus ambiguous noun types (see Sá‐Leite & Lago, 2024) within comprehension and memory paradigms.

Finally, it is also important to acknowledge the limitations of our study, such as the presence of the “surzhyk” dialect in the Ukrainian language. “Surzhyk” is an oral, nonstandard mixed idiom that involves a blend of Ukrainian and Russian languages, and its usage could lead to mislabeling the grammatical gender of objects (Kostiučenko, 2023). To our knowledge, it is not possible to detect the usage of this dialect using proficiency tests in Ukrainian or Russian. It can only be observed in oral communication or if a participant reports it in their linguistic profile.

To conclude, potential limitations notwithstanding, bilingual participants with two distinct three‐gendered grammatical systems, but not monolingual speakers of the genderless language, showed a grammatical gender effect (i.e., better recall for names congruent with the object's grammatical gender). This suggests that the effect of language on mental representations of objects might be observed even in speakers of two three‐gendered languages when those objects have matching grammatical gender in both of those languages. However, proficiency did not modulate the grammatical gender effect when the objects had contrasting grammatical genders across Ukrainian and Russian. This indicates that the grammatical gender of the most proficient language of a simultaneous bilingual did not affect mental representations when it mismatched the less proficient language. Rather, the likelihood of a three‐gendered grammatical system influencing memory recall may rest on the mechanisms by which top‐down retrieval is facilitated, such as gender congruency and cognate status across languages.

## Supporting information



Supplementary Information

## Notes

1 We thank Reviewer 1 for suggesting this interpretation.

2 We thank Reviewer 2 for this interpretation.
