# Supplementary material for: Between Two Grammatical Gender Systems: Exploring the Impact of Grammatical Gender on Memory Recall in Ukrainian−Russian Simultaneous Bilinguals
Source: Cogn Sci. 2025 Oct 2;49(10):e70117. doi: 10.1111/cogs.70117 (PMC12490866; doi:10.1111/cogs.70117)
Supplement: Supplementary file 1 — Supplementary Information [file COGS-49-e70117-s001.docx]

**Supplementary materials**

In addition to analysing accuracy, we also explored reaction times to investigate whether there was any significant difference between Ukrainian-Russian bilinguals and English monolinguals, as well as within the Ukrainian-Russian bilingual group based on language proficiency. For this, we constructed linear mixed-effects models with reaction time as the dependent variable. Specifically, for the first part of the analysis, we modelled reaction times as influenced by the interaction between the condition (Congruent in both L1s vs Incongruent in both L1s) and participant group (bilingual vs. monolingual). Random intercepts and random slopes were included for the interaction between condition and group for both participant and item. In the second part of the reaction time analysis, we also examined whether reaction times within the Ukrainian-Russian bilingual group were influenced by the L1 proficiency. We modelled reaction times as a function of the interaction between language proficiency and condition (Congruent in Russian & Incongruent in Ukrainian vs. Congruent in Ukrainian & Incongruent in Russian), including random intercepts and random slopes for the interaction for both participant and item.

**Figure S1.**

*Reaction Times by Condition (Congruent in both L1s vs Incongruent in both L1s) and Participant Group (English Monolinguals vs. Ukrainian-Russian Bilinguals)*


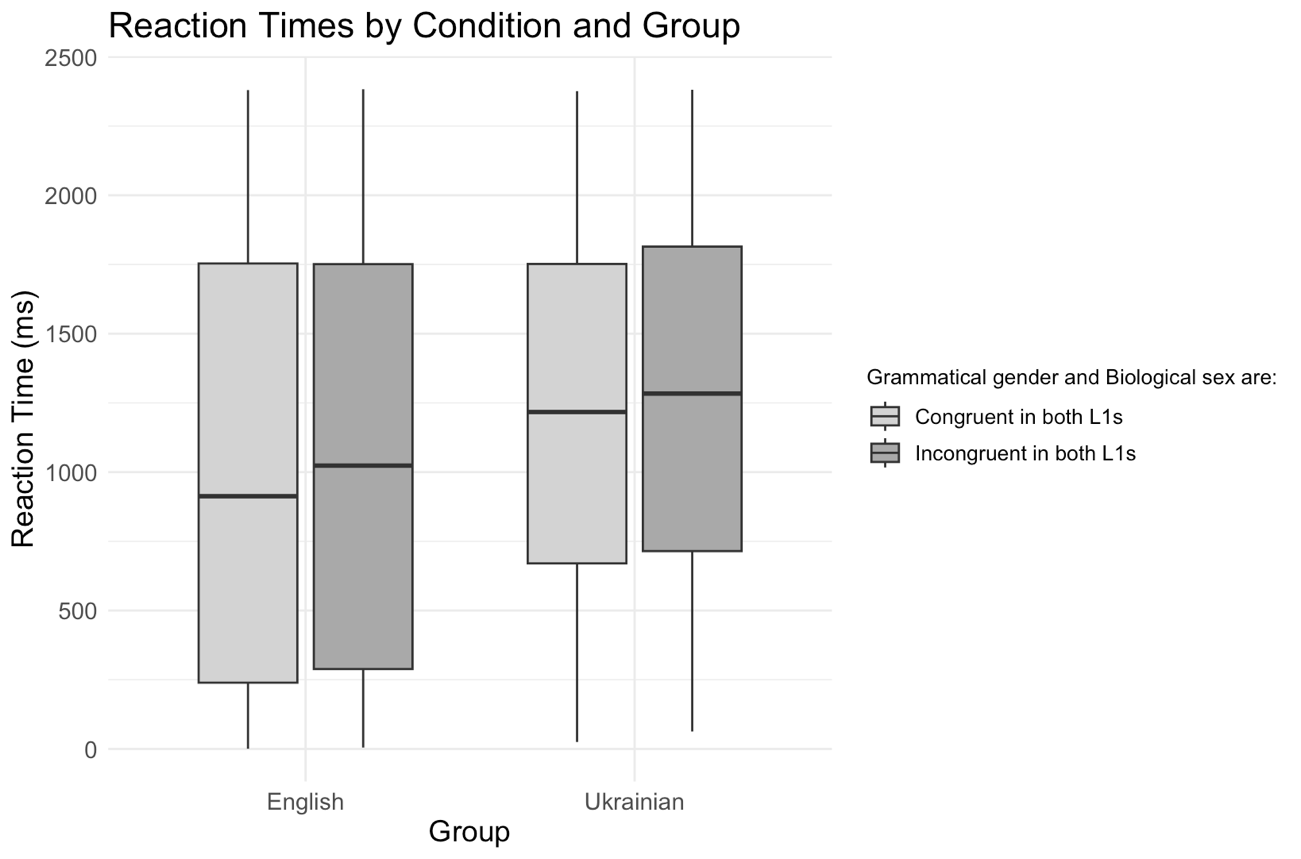


However, when analysing reaction times for the responses, the difference between Ukrainian-Russian bilinguals and English monolinguals was nonsignificant (figure S1). Specifically, the reaction times (RT) for bilingual participants were somewhat slower but not significantly different from those of monolinguals (*Estimate = -26.92,* *SE = 176.49, t =-0.15, p = .879)*. This indicates that while accuracy was influenced by the congruency between grammatical gender and biological sex, this congruency did not lead to faster response times. Main effects of Condition and Group were also non-significant (Condition: *Estimate = -41.62,* *SE = 193.12, t =-0.22, p = .832;* Group*: Estimate = 136.04,* *SE = 275.76, t =0.49, p = .623).* Additionally, when looking at bilingual group alone, there was no significant difference in reaction times based on the condition (*Estimate = -49.53,* *SE = 183.11, t =-0.27, p = .790)*.

**Figure S2.**

*Reaction Times by Condition (Congruent in Ukrainian & Incongruent in Russian vs Congruent in Russian & Incongruent in Ukrainian) and Language Proficiency (Ukrainian-Russian Bilinguals)*


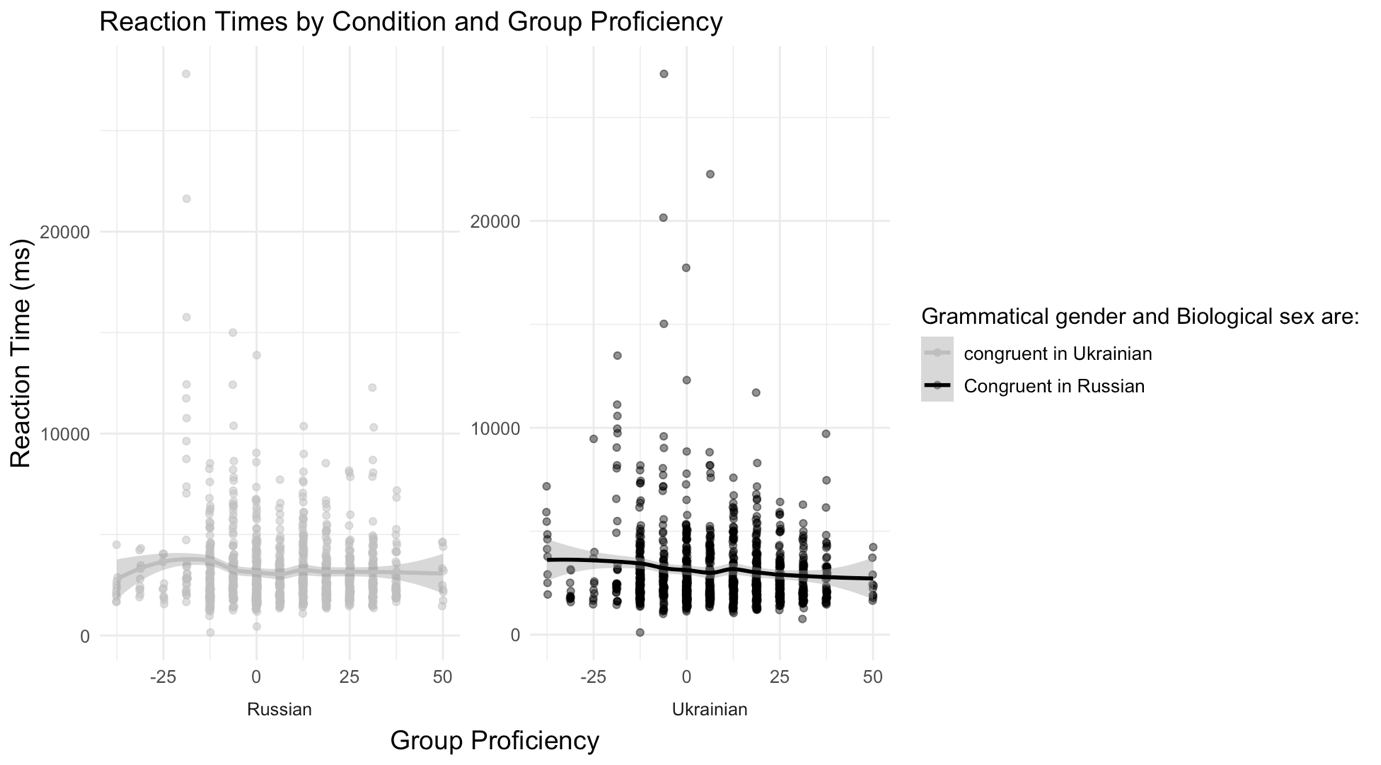


Similarly, as can be seen in figure S2, the results showed no significant difference in RTs based on the interaction between group proficiency and the condition (*Estimate = -3.73. SE = 4.64, t = -0.80, p = .422*). Main effects of condition and group proficiency were also non-significant (Condition: *Estimate = -119.00,* *SE = 171.04, t =-0.70, p = .495;* Group Proficiency*: Estimate = -11.15,* *SE = 7.15, t =-1.56, p = .122).* These findings suggest that language proficiency did not significantly impact the speed of responses, consistent with the lack of effect on recall accuracy.
